# AI assisted focused cardiac ultrasound in preventive cardiology – a perspective

**DOI:** 10.1038/s44325-025-00063-9

**Published:** 2025-06-26

**Authors:** Ido Cohen, Adi Lakritz, Elad Maor

**Affiliations:** 1 Leviev Cardiovascular Institute, Sheba Medical Center, Ramat Gan, Israel; 2 Faculty of Medical and Health Sciences, Tel Aviv University, Tel Aviv, Israel; 3 Aisap.ai, Ramat Gan, Israel

**Keywords:** Cardiovascular diseases, Medical imaging, Ultrasonography, Echocardiography

## Abstract

Integrating focused cardiac ultrasound (FoCUS) and AI transforms cardiovascular health care. We advocate for the application of these technologies for the early detection and management of various cardiac conditions, expanding beyond traditional atherosclerotic cardiovascular disease detection. In this perspective, we propose a framework that utilizes AI-assisted FoCUS to assess biological age as a risk factor and identify valvular heart disease, occult atrial fibrillation, heart failure, and pulmonary hypertension.

## Introduction

Cardiovascular disease (CVD) is the leading cause of morbidity and mortality worldwide^1–3^. Current forecasts indicate that the age-standardized prevalence of global CVD is projected to remain stable, reflecting a plateau in the effectiveness of existing prevention strategies^4,5^. Efforts in preventive cardiology predominantly target atherosclerotic cardiovascular disease (ASCVD). Fundamental measures, such as smoking cessation and lifestyle modifications, are universally recommended to reduce cardiovascular risk across the population. More aggressive interventions, including lipid-lowering therapies, antihypertensives, antiplatelet agents, and anti-inflammatory medications, have established roles in cardiovascular risk management. However, critical questions remain: which individuals should receive these interventions? What are the appropriate thresholds for intervention, and what target values and parameters should be followed? Guidelines seek to address this by categorizing patients into distinct risk groups and utilizing risk calculators that consider factors such as age, sex, smoking status, blood pressure, and cholesterol levels^6,7^. Although these risk prediction tools are widely accepted, identifying individuals who would most benefit from intensive interventions remains challenging. These tools often overestimate risk in older adults, underestimate it in younger populations, and may miscalculate risk across diverse demographic groups. Moreover, they frequently show reduced sensitivity, failing to account for all potential cardiovascular events, raising concerns about their reliability and effectiveness in guiding preventive strategies^8,9^. As current risk estimation approaches remain imperfect, there is a substantial opportunity to enhance ASCVD prevention through the incorporation of risk modifiers—demographic, clinical, laboratory, and imaging parameters—that can refine traditional risk assessments. However, evidence-based risk modifiers are limited, and those that are well-known may not be accessible or affordable for the entire population. Thus, the quest for simple and accessible risk modifiers continues, with a focus on their potential to improve cardiovascular outcomes.

As the landscape of preventive cardiology evolves, a paradigm shift is essential to broaden the focus beyond ASCVD to encompass a wider array of cardiac conditions, including heart failure (HF), diastolic dysfunction, valvular heart disease (VHD), arrhythmias, and pulmonary hypertension (PH). These conditions often present subclinical phases that are particularly amenable to targeted preventive efforts, allowing for timely follow-up and intervention before clinical symptoms manifest or at least before the complete clinical syndrome develops. Furthermore, the application of established pharmacological and instrumental strategies at early stages of these conditions can significantly enhance outcomes and promote overall cardiovascular health.

**Fig. 1:**
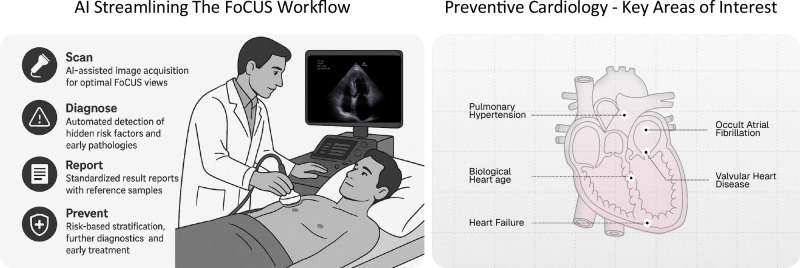
AI-assisted focused cardiac ultrasound in preventive cardiology. The central figure illustrates the integration of AI into the workflow of focused cardiac ultrasound (FoCUS) to advance preventive cardiology. The left panel describes the sequential AI-enhanced workflow: Scan—AI facilitates optimal image acquisition tailored to focused cardiac views; Diagnose—automated identification of subclinical risk factors and early cardiac pathologies; Report—standardized reporting enhanced by reference samples for improved consistency; and Prevent—risk stratification informing further diagnostic evaluation and personalized therapeutic interventions. The right panel highlights key areas of interest in preventive cardiology where AI-driven applications hold transformative potential, including biological cardiac age, valvular heart diseases, occult atrial fibrillation, heart failure, and pulmonary hypertension.

In pursuit of promoting cardiovascular health, the integration of new technologies and various imaging modalities into clinical practice is continually being explored. Among these modalities, focused cardiac ultrasound (FoCUS) is a clinical imaging tool whose value can be substantially enhanced by Artificial Intelligence (AI). This approach, as will be discussed in this perspective article, may facilitate early and reliable detection of cardiovascular pathology across its spectrum, thus providing better assessment of individual patient risk and guidance for the timing of intervention, prevention, and health promotion (Fig. 1).

## Focused cardiac ultrasound (FoCUS) and AI-assisted FoCUS

FoCUS refers to the rapid acquisition, interpretation, and immediate clinical integration of cardiac ultrasonographic imaging performed by the treating clinician^10^. While the term is not defined by the exam’s location, the capabilities of the imaging device, or the practitioner’s specialty, FoCUS is commonly used at the bedside or in outpatient settings, often with handheld portable devices operated by non-cardiologists or non-sonographers^11,12^, thereby democratizing access to ultrasound technology in healthcare, particularly in underserved area^13–16^. Furthermore, FoCUS offers the advantage of repeatability, allowing for consistent monitoring of cardiac conditions without the concerns associated with radiation exposure, making it a safe and effective tool for patient evaluation^17^.

These advantages of FoCUS, combined with its capacity to provide critical, often life-saving information in emergency settings, have contributed to its widespread adoption in critical care and emergency disciplines—representing the most recognized and endorsed applications by medical societies to date^18–23^. As a versatile tool, the use of FoCUS has expanded beyond acute scenarios, with physicians increasingly incorporating it into routine assessments as an adjunct to the physical exam, a practice known as the ultrasound-assisted physical exam^10,24–26^. This broader application enhances diagnostic accuracy and fosters more informed clinical decision-making across various healthcare settings.

It is essential to acknowledge that the inherently limited approach associated with FoCUS does not imply substandard imaging; methodologies have been developed to establish end-to-end standardization in the field^27,28^. To address the potential risks posed by the increasing number of less experienced operators performing these tests, comprehensive training guidelines and curricular recommendations have been established^29,30^. These guidelines underscore the importance of academic coursework, online resources, simulation training, and practical experience in scanning, all of which are evaluated based on the quality of scans and total training time. Such measures aim to prepare practitioners adequately for effective performance and interpretation of scans. From a broader systemic perspective, FoCUS workflow processes have emerged to contextualize the examination as a formal procedure within clinical practice. This encompasses diagnostic cascades, imaging storage, and formal report generation, as well as quality control processes related to image acquisition and interpretation, and reimbursement mechanisms^27,28,31–34^.

A significant revolution in the healthcare industry is the rapid advancement of AI, which has transformed the landscape of cardiac imaging^35–37^. The integration of AI into FoCUS has resulted in AI-assisted FoCUS, a novel approach addressing existing challenges in the field and effectively improving processes^38–40^. In image acquisition, AI enhances operators’ ability to capture high-quality ultrasound images by providing probe navigation guidance or real-time grading of views, thereby reducing imaging exclusions and decreasing scanning time^41–44^. Moreover, AI facilitates the interpretation and analysis of acquired images by providing automatic raw measurements, reporting clinically significant diagnoses, and utilizing big data analysis to reveal insights that may not be readily identified even by experts, thereby promising to reduce interpretative omissions and misinterpretations^45–47^. Once the examination concludes, AI can automatically generate formal reports and distribute findings to relevant personnel in clinical settings, also facilitating future recommendations^48^. These functionalities mitigate operator dependency, enabling a wider range of healthcare professionals to utilize cardiac ultrasound efficiently. The synergistic effects of AI-assisted FoCUS are poised to enhance diagnostic accuracy and streamline workflows, ultimately improving patient outcomes and raising the standard of care in cardiology.

## AI-assisted FoCUS in promoting cardiovascular health

We propose that routine FoCUS and AI-assisted FoCUS be utilized in screening scenarios to assess five key areas: biological cardiac age as an ASCVD risk modifier, VHD, occult atrial fibrillation (AF), early HF, and PH.

### Biological cardiac age as an ASCVD risk modifier

Chronological age is a significant driver of CVD risk^49^. Nevertheless, it may not always accurately reflect an individual’s overall health or aging process. As individuals age and experience the onset of diseases at varying rates, the concept of biological age has emerged as a compelling alternative, emphasizing an individual’s physiological state shaped by genetics, lifestyle, and environmental influences^50,51^. Incorporating biological age, and not chronological age, into contemporary ASCVD risk calculators holds the potential to significantly improve their accuracy.

Biological cardiovascular aging exemplifies this concept; it is a complex and non-linear process characterized by a highly variable relationship with chronological age. As individuals grow older, the heart undergoes a series of phenotypic alterations, including changes in structure, function, and vascular elasticity, which may not always correspond to the expected outcomes of chronological aging. Therefore, understanding biological cardiac age within the context of cardiovascular health holds great potential^52,53^. To estimate biological age, the fields of longevity science and biohorology prioritize the identification of aging biomarkers—measurable parameters that consistently change, both qualitatively and quantitatively, with human aging. These biomarkers facilitate the prediction of an individual’s aging trajectory, whether it is accelerated or delayed. Ideal aging biomarkers correlate with chronological age in healthy individuals, provide more accurate predictions of adverse outcomes, reveal the early stages of age-related diseases, and enable minimally invasive assessments^54^.

Among various clinical and functional tests that serve as biomarkers for cardiovascular aging, simple, non-invasive cardiac imaging techniques may be effective indicators for assessing cardiac aging trajectories. In the field of echocardiography, recognized remodeling patterns associated with aging include changes in myocardial mass, a decline in ventricular volumes, reduced resting left ventricular systolic function, gradual declines in left ventricular strain, diastolic dysfunction, and an increase in left atrial volume^55^.

The SardiNIA trial^56^, conducted in Italy with 2,614 healthy subjects, demonstrated that these echocardiographic metrics can not only predict biological age but also have significant clinical implications for cardiovascular health management. The study utilized comprehensive echocardiographic measurements to define heart aging patterns. An accelerated heart aging trajectory was observed in ~15% of patients and was associated with a significant elevation in the primary endpoint of the study, which included both fatal and non-fatal cardiovascular events (HR 2.00 [1.14–3.51], *p* value = 0.01). However, despite the compelling nature of these results, practical application presents significant challenges. The implementation of such a model requires formal echocardiographic acquisition and the measurement of designated metrics, which must be benchmarked against normative population distributions. This process is time- and resource-consuming and is further hindered by issues of availability and affordability. These limitations restrict the widespread utilization of such tools in cardiac prevention.

The solution may reside in the advancements driven by the machine learning and big data revolution. Faierstein et al.^57^ provided proof of concept that machine learning models based on echocardiography scans can estimate biological age. Significant discrepancies from chronological age have prognostic implications for overall survival, depending on whether biological age is perceived as older or younger. The aforementioned study, conducted in Israel, utilized 120,127 echocardiography scans from a single tertiary hospital. Notably, patients whose estimated age exceeded their chronological age by 5 years or more had an independent and significant 34% increased risk of death during follow-up (95% CI, 1.22–1.46; *P* < 0.001). In contrast, patients classified as being 5 years or more younger than their chronological age were 13% less likely to experience death during the follow-up period (HR, 0.87; 95% CI, 0.84–0.91; *P* < 0.001). Importantly, this model underwent external validation in a prospective cohort where FoCUS exams were performed by non-cardiologist physicians in medical wards. The evaluation utilized a data set of prospectively enrolled patients who were randomly scanned using Philips Lumify handheld devices during their hospitalization. The final cohort consisted of 319 point-of-care examinations with consistent results with respect to accuracy of biological age prediction (MAE of 6.6 years, an RMSE of 8.3 years, and a Pearson correlation coefficient of 0.91).

These findings illustrate that machine learning algorithms can effectively leverage FoCUS to estimate biological age, enhancing cardiovascular risk stratification beyond current models. Consequently, when a patient visits a primary physician or screening program, a FoCUS scan can deliver an estimated biological age within minutes. This estimated age can be utilized to calculate delta age, serving as a critical risk modifier, or it can replace chronological age in standard risk calculators, facilitating more personalized and timely patient management.

### Valvular heart disease

The global incidence of VHD has risen significantly over the past 30 years, largely due to the increasing prevalence of degenerative valvulopathies associated with an aging population^58^. Two prospective cohort studies, HONU Valve^59^ in the USA and OxVALVE^60^ in the UK, assessed the underdiagnosis of VHD in elderly populations (aged 65 and above) without a prior diagnosis. The HONU study found that 16% of participants had clinically significant valvulopathy (moderate to severe), while the OxVALVE study diagnosed only 6.4% with clinically significant valvulopathy. Early detection of VHD is crucial, as it enables timely intervention for both symptomatic and severe asymptomatic patients, improving prognosis and reducing cardiac complications such as HF and arrhythmias, which result from irreversible cardiac remodeling and permanent myocardial damage. This underscores the need for enhanced screening strategies.

To integrate FoCUS as a screening modality for VHD, a study conducted by the Mayo Clinic from 2017 to 2022 demonstrated a strong agreement between FoCUS and transthoracic echocardiography (TTE), revealing high sensitivity and specificity for detecting moderate to severe aortic stenosis, aortic regurgitation, mitral regurgitation, and tricuspid regurgitation (TR)^61^. Concurrently, studies utilizing AI have shown the capability to accurately diagnose VHD based on comprehensive TTE images^62–65^, suggesting that the combination of FoCUS and AI may enhance the efficacy of VHD screening.

In examining the proof of concept for AI-assisted FoCUS technology, a prospective trial at Sheba Medical Center in Israel assessed its application in primary medical evaluations, wherein non-cardiologist trainees utilized an AI-enhanced device to screen patients within the emergency department and internal medicine wards. While the prevalence of valvular disease in this research population does not reflect outpatient scenarios, it serves as a proof of concept for the technology, as 10% of patients were diagnosed with previously unknown significant valvular disease^66^.

### Screening for occult atrial fibrillation

AF is the most common arrhythmia worldwide, with its overall prevalence steadily increasing^67,68^. Given that AF is associated with multiple adverse outcomes—including death, stroke, HF, and recurrent hospitalizations—new management approaches are essential in this field^69–71^. Underdiagnosis of AF is a significant concern, with current estimates suggesting that 10–30% of cases remain undetected. This issue arises in part because AF can be asymptomatic and paroxysmal, often evading recognition during routine physical examinations and ECG testing. Importantly, patients with occult AF may present with the same complications as those with overt AF, highlighting the critical need for proactive measures to identify this subset of the patient population^72–74^.

This concern has heightened interest in population screening for AF^75,76^. Currently, population risk stratification for screening predominantly hinges on age and stroke risk factors—an approach that lacks nuance. Improving these strategies could significantly enhance the identification of individuals at risk. Leveraging AI and big data to analyze non-invasive cardiac data may facilitate the identification of patients at risk for occult AF, leading to more precise screening recommendations. Given that AF is associated with structural changes in the heart—especially concerning left atrial function and remodeling^77–79^—it is posited that applying machine learning algorithms to echocardiographic scans in sinus rhythm could effectively uncover patients with undiagnosed AF.

In research conducted by Yuan et al.^80^, an AI model based on comprehensive echocardiography parasternal long axis views was able to predict concurrent paroxysmal AF with an AUC of 0.75, a sensitivity of 69%, a specificity of 68%, and an overall accuracy of 68%. Notably, this performance reinforces the superior diagnostic capabilities of AI-driven echo-based algorithms, surpassing AUCs of up to 0.64 seen in traditional risk factor-based models and individual echocardiographic measurements. Furthermore, in a subgroup of patients, the evaluation of TTEs paired with ECGs revealed that TTE significantly contributed to the final combined ensemble prediction of AF, outperforming ECGs alone. Although this study has not yet explored the model’s utility in FoCUS, the results, derived from echocardiograms captured with a singular view and without the need for complex measurements or specialized technical skills, suggest significant potential for broader application in FoCUS settings.

These findings illuminate a vision where AI-assisted FoCUS could play a role in identifying patients with undiagnosed AF. Utilizing such algorithms may provide the nuanced insights necessary to pinpoint the subset of patients who would benefit most from prolonged monitoring, including Holter tests and loop recorder implementations. By identifying cases of undiagnosed AF and initiating anticoagulation for eligible patients, it may be feasible to reduce the burden of stroke and improve overall management outcomes.

Another promising utilization of this tool could be to enhance primary prevention efforts for AF. Even in scenarios where AF is not definitively diagnosed, identifying echocardiographic characteristics consistent with AF may indicate the presence of an “arrhythmogenic heart” that warrants specialized preventive care. Maintaining a healthy lifestyle—comprising regular physical activity, avoiding excessive alcohol intake, and managing weight and blood pressure, alongside adherence to HF guideline-directed medical therapy—has been shown to effectively reduce the risk of AF. Individuals categorized by the model as having a higher probability of developing AF may benefit from more rigorous guidance and tailored interventions aimed at mitigating their risk.

### Early detection of heart failure

HF is a complex clinical syndrome that leads to significant morbidity and mortality, with its prevalence continuing to rise due to an aging population and increasing risk factors^81^. Often beginning as a subclinical condition, HF progresses gradually from an asymptomatic stage characterized by structural or functional dysfunction, making early diagnosis critical. Identifying HF in these early stages is essential, as effective treatments across the spectrum of ejection fractions and for specific etiologies are now available, which can significantly change patient prognosis^82,83^. As echocardiography is the primary diagnostic modality for diagnosing, classifying, and identifying specific etiologies of HF^84^, we propose that AI-assisted FoCUS could enhance early diagnosis and treatment of this condition.

AI-assisted FoCUS has shown significant capabilities in diagnosing both systolic and diastolic dysfunctions. In diagnosing systolic dysfunction, AI-assisted FoCUS effectively evaluates global left ventricular ejection fraction (LVEF), yielding results comparable to established standards, with sensitivities and specificities ranging from 85% to 90%^85–87^. Research by Dadon et al. highlights the prognostic implications of AI-assisted LVEF assessment, linking lower ejection fractions to increased 1-year mortality and rehospitalization^88,89^. Additionally, regional wall motion abnormalities (RWMA) have been successfully evaluated through AI-assisted FoCUS^90,91^; though primarily associated with ischemic heart disease, their potential utility in screening asymptomatic populations is noteworthy. For instance, a study by Espersen et al. ^92^ involved 3415 asymptomatic participants and found that RWMA were identified in 2.4% of cases, which were associated with a significant risk of incident HF, independent of other clinical factors (HR 3.63, *P* < 0.001). Furthermore, AI plays a significant role in assessing diastolic dysfunction. Research by Chen et al.^93^ found that AI analysis of comprehensive TTEs effectively evaluates diastolic dysfunction. Notably, the study demonstrated that even with videos from a single apical four-chamber view without Doppler capabilities, an AUC of 0.88 was achieved for distinguishing normal/intermediate diastolic function from diastolic dysfunction, and an AUC exceeding 0.9 for diastolic grading, indicating potential for further utilization in FoCUS settings.

Furthermore, AI-assisted FoCUS holds promise in detecting precursor states of HF as well as identifying underlying diagnoses. One crucial precursor is left ventricular hypertrophy, which has been shown in recent research by Firma et al.^94^ to be effectively diagnosed using AI-assisted FoCUS, particularly in underserved areas. Additionally, rarer etiologies of cardiomyopathies, such as Hypertrophic Cardiomyopathy and Amyloidosis, can also be identified through AI models utilizing comprehensive TTEs^95,96^ and FoCUS^97^. While designated broad screening for these rare diseases may not be cost-effective, AI models can leverage images acquired for other clinical purposes to diagnose these conditions as incidental findings, presenting a notable advantage in clinical practice.

### Pulmonary hypertension

PH affects up to 1% of the global population, with 10% of individuals aged 65 and older impacted^98^. Despite these prevalence rates, routine screening for PH remains limited, leading to underdiagnosis and delayed detection, often until the disease has progressed to advanced stages. Early detection of PH is critical, as novel treatments are now available that can significantly improve outcomes^99^. The gold standard for diagnosing PH is right heart catheterization, but its invasive nature limits its frequency of use. Consequently, comprehensive TTE has become an essential non-invasive tool for initial screening^100,101^.

The current FoCUS screening methods for PH predominantly rely on measurements of peripheral veins, particularly the inferior vena cava, to estimate right atrial pressure^102^. In addition to this method, research has indicated that novice sonographers can measure TR jet velocity using devices equipped with continuous wave Doppler. This measurement can be utilized to assess right ventricular systolic pressure^103^. Theoretically, combining these two assessments could enable a more comprehensive estimation of PH. However, practical implementation remains relatively complicated due to the advanced training required and the necessity for Doppler capabilities within the ultrasound probe to accurately assess TR jet velocity.

While there is no published data on AI-assisted FoCUS for diagnosing and classifying PH, we hypothesize it could be a significant application. This is based on the ease of identifying the right ventricle, tricuspid valve, and inferior vena cava using standard FoCUS views, existing literature on echocardiographic signs of RV dysfunction and PH, and AI’s potential to integrate multiple parameters for improved decision-making. Additionally, machine learning algorithms could simplify evaluations by reducing reliance on extensive raw data, minimizing the need for strain and Doppler techniques, and streamlining the diagnostic process.

## Clinical implications and future perspectives

In this review, we present a vision for promoting cardiovascular health through the integration of AI-assisted FoCUS. This approach signifies multiple paradigm shifts: from the utilization of FoCUS primarily in emergency and critical care settings to its application in preventive cardiology; from an exclusive focus on ASCVD to a more holistic care strategy; and from predominantly human-based FoCUS capabilities to innovative AI-assisted technologies. In line with this vision, apparently healthy adults will undergo systematic screening within primary care environments. Primary care providers will actively conduct AI-assisted FoCUS examinations during these encounters, enabling automatic assessments of biological age that contribute to improved ASCVD risk estimations, evaluations of VHD, screenings for occult AF, and assessments of subclinical HF and PH.

It is imperative to acknowledge that the practical implementation of AI-assisted FoCUS in preventive cardiology faces significant challenges. Much of the existing evidence stems from theoretical studies that demonstrate proof of concept but have yet to be commercialized or integrated into clinical practice. Currently, individual models have been evaluated in isolation, creating barriers to the integration of multiple diagnostic assessments. Although a single scan has the capacity to yield data across various clinical domains, most research has not explored the simultaneous evaluation of these integrated models. Consequently, a significant challenge lies in merging disparate models to facilitate concurrent analysis within a singular diagnostic framework.

Furthermore, FoCUS is typically performed using handheld probes, which may present limitations such as poor image quality and a lack of advanced features like spectral Doppler capabilities. As a result, AI models need to be adapted specifically for the FoCUS context, concentrating on delivering outcomes based solely on 2D and color Doppler images produced by handheld devices. This necessitates either the creation of new models or external validation for FoCUS exams, which is often overlooked.

Additionally, given the visionary nature of this proposal, there are currently no clinical trials demonstrating its efficacy. Prospective research is necessary to evaluate its impact on clinical outcomes. The population that may benefit from such interventions is yet to be characterized, and it is plausible that not the entire adult population necessitates the comprehensive assessments delineated above. Rather, these assessments could be tailored, with distinct demographic groups potentially benefiting from different modalities. For instance, younger adults may derive greater benefit from biological cardiac age estimation and the diagnosis of cardiomyopathies, while older adults may gain more from the identification of valvular disease and screening for occult AF.

Questions also arise regarding the operators involved in implementing such protocols. To extend its reach to a broader population, the demographic engaged in scanning must be expanded to include primary care physicians and potentially other healthcare providers, such as physician assistants and registered nurses. To facilitate this expansion, dedicated training programs should be developed to address the needs of this new cohort of operators, focusing on specific scanning targets aimed at enhancing cardiovascular health, as well as imparting an understanding of the advantages and limitations of the modalities.

Another critical aspect is the development of efficient workflows for integrating AI-assisted FoCUS into clinical practice. Standardized processes are essential to optimize patient outcomes. Formal reports with images, operator impressions, and AI interpretations should be saved and stored securely for quality assurance, medico-legal aspects, and billing purposes. Positive findings should lead to referrals for comprehensive TTE and cardiologist follow-up. At this stage, negative results must be interpreted cautiously, and it is vital to consider the entire clinical picture, including patient history, physical examination, and results from other diagnostic modalities, to avoid misinterpretations or missed diagnoses due to overreliance on AI-assisted FoCUS.

In conclusion, the integration of AI-assisted FoCUS has the potential to streamline cardiovascular assessments, improve the early detection of cardiac conditions, and enhance patient outcomes through timely interventions. The realization of this vision will require collaborative efforts among researchers, clinicians, and technology developers to address the barriers of implementation, standardization, and accessibility in diverse healthcare settings. As advances in technology continue to evolve, embracing this comprehensive strategy will be crucial for addressing the growing burden of CVD and improving overall public health.

## Supplementary information

Supplementary Table

## Data Availability

No datasets were generated or analyzed during the current study.

## Abbreviations

AF: Atrial fibrillation
AHA/ACC: American Heart Association/American College of Cardiology
AI: Artificial intelligence
AR: Aortic regurgitation
AS: Aortic stenosis
ASCVD: Atherosclerotic cardiovascular disease
AUC: Area under the curve
CI: Confidence interval
CVD: Cardiovascular disease
ECG: Electrocardiography
FoCUS: Focused cardiac ultrasound
HF: Heart failure
HFpEF: Heart failure with preserved ejection fraction
HFrEF: Heart failure with reduced ejection fraction
HR: Hazard ratio
LVEF: Left ventricular ejection fraction
MAE: Mean absolute error
MR: Mitral regurgitation
PH: Pulmonary hypertension
PLAX: Parasternal long-axis
RMSE: Root mean square error
RWMA: Regional wall motion abnormality
TR: Tricuspid regurgitation
TTE: Transthoracic echocardiographic
UAPE: Ultrasound-assisted physical exam
VHD: Valvular heart disease
