# Supplementary material for: AI assisted focused cardiac ultrasound in preventive cardiology – a perspective
Source: NPJ Cardiovasc Health. 2025 Jun 26;2:27. doi: 10.1038/s44325-025-00063-9 (PMC12912408; doi:10.1038/s44325-025-00063-9)
Supplement: Supplementary file 1 — Supplementary Table [file 44325_2025_63_MOESM1_ESM.pdf]

Supplementary Table: Study Cohort, Examiner Profiles, and Echocardiographic Techniques from Referenced Studies

Reference #	Prevention key area	Study	Study Cohort	Examiner Profile	Echocardiographic Techniques and Equipment
56	Biological cardiac age	SardiNIA trial	2,614 healthy subjects ranging in age from 18 to 93 years old	An experienced echocardiographer	Comprehensive TTE with Doppler (ATL HDI 3500, Ultramark Inc.; Acuson X300, Siemens Healthineers)
57	Biological cardiac age	Faierstein et al.	120,127 patients aged over 18 years	Retrospective cohort - echocardiography lab sonographers<br>Prospective cohort- Physicians after short training course for POCUS	Retrospective cohort - point-of-care examinations to examinations conducted in the echo-cardiography laboratory using 20 different types of ultrasound devices.<br>Prospective cohort -Philips Lumify handled POCUS device<br>aisap.ai AI model
59	Valvular heart disease	HONU valve study	928 patients ≥65 year old without a prior diagnosis of VHD	A board-certified echocardiologist or an accredited sonographer	Comprehensive TTE
60	Valvular heart disease	OxVALVE study	2,500 individuals ≥65 year old without prior diagnosis of VHD	British Society of Echocardiography accredited sonographer	Comprehensive TTE

61	Valvular heart disease	Wen et al.	77 patients with an average age of 72	POCUS - experienced physician in a general cardiology clinic<br><br>Standard TTE - conducted by registered cardiac sonographers and interpreted by cardiologist	Bedside POCUS -Philips CX50 portable ultrasound machine, equipped with two-dimensional (2D) B-mode, M-mode, color Doppler, pulsed-wave (PW) and continuous-wave (CW) Doppler, along with an S5 1-MHz phase-array transducer featuring a resolution of 1400 × 1050 pixels.<br><br>Standard TTE - Vivid E9 (2017–2019) and E95 (2019 to 2022); GE Vingmed Ultrasound, Horten, Norway
62	Valvular heart disease	Krishna H et al.	256 patients with an average age of age of 67	Trained sonographers	Commercially available ultrasound systems (Philips, GE, Siemens).<br><br>Us2.ai AI model
63	Valvular heart disease	Vrudhula et al.	Training set - 47,312 studies from Cedars-Sinai Medical Center<br><br>Test set - 2,462 studies from Cedars-Sinai Medical Center, 5,549 studies from Stanford Healthcare<br><br>Population characteristic are not available	Echocardiography lab sonographers	Comprehensive TTE with color doppler<br><br>Non commercial AI model

64	Valvular heart disease	Vrudhula et al.	Training set - 58,614 studies from Cedars Sinai medical Center<br>Test set - 1800 studies from Cedars-Sinai Medical Center , 915 studies from Stanford Healthcare	Echocardiography lab sonographers	Comprehensive TTE with color doppler<br><br>Non commercial AI model (PyTorch Lightning deep learning framework)
65	Valvular heart disease	Sadeghpour et al.	Test set - 438 patients, average age 73, based on PROMISE and COAPT trials cohorts	Echocardiography lab sonographers	Comprehensive TTE with color doppler<br><br>Us2.ai AI model
66	Valvular heart disease	Fisher et al.	659 patients with mean age of 66	Medical interns and residents	Philips Lumify POCUS devices<br><br>aisap.ai AI model
80	Atrial fibrillation	Yuan et al.	111,319 TTE studies	Echocardiography lab sonographers	Comprehensive TTE - Philips EPIQ 7 or iE33 ultrasound machine -<br><br>Non commercial AI model based on PLAX view without Doppler imaging

85	Heart Failure	Motazedian P. et al.	424 patients with median age of 65	AI assisted FoCUS - Senior and novice (<100 scans) sonographers<br><br>Ground truth - trained cardiac sonographer	AI assisted FoCUS - EchoNous KOSMOS point-of-care ultrasound machine device and AI algorithm. Based on A4C and A2C views with no doppler.<br><br>Ground truth - comprehensive TTE (cart based machines)
86	Heart Failure	Huang W. et al.	100 patients with suspected HF, mean age 61	AI assisted FoCUS - novice sonographers with 2 weeks of training<br><br>Ground truth - trained sonographers interpreted by cardiologists	AI assisted FoCUS- data acquisiton - EchoNous Kosmos handheld echo data interpretation - US2.AI<br><br>Ground truth - comprehensive echocardiography
87	Heart Failure	Kagiyama N. et al.	200 patients in two Japanese hospitals	AI assisted FoCUS - cardiologist<br><br>Ground truth - clinical sonographer	AI assisted FoCUS - EchoNous handheld device with automated EF analysis system. Based on A4C and A2C views with no doppler.<br><br>Ground truth - comprehensive echocardiography
88,89	Heart Failure	Dadon et al.	88 patients with mean age of 58	Eight students underwent a 6-hour didactic and hands-on training session	Hand- held ultrasound device equipped with an AI based tool for automatic calculation of LVEF from the A4Cv view.

90	Heart Failure	Lin, X. et al.	6,953 examinations from 2 medical centers	Echocardiography lab sonographers	Comphrensive echocardiography machine manufacturers including Phillips EPIQ 7C and iE-elite with S5-1 and X5-1 transducers (Phillips, Andover, MA, United States), and Vivid E95 (General Electric, Fairfield, CT, United States) and portable bedside machines including Philips CX50 and Mindray M9cv with transducer SP5-1s (Mindray, Shenzhen, Guangdong, China).<br><br>Non commercial AI algorithm
91	Heart Failure	Slivnick, J. et al.	15,746 TTE studies	Echocardiography lab sonographers	Comphrensive echocardiography - Philips ultrasound imaging equipment<br><br>Non commercial AI algorithm
92	Heart Failure	Espersen et al.	3,415 participants without prior diagnosis of heart failure or ischemic heart disease	3 experienced sonographers	Comphrensive echocardiography - Vivid 5 ultrasound machines (GE Healthcare, Horten, Norway) with a 2.5 MHz transducer
93	Heart Failure	Chen et al.	Test set - 388 prospective studies	Expert sonographers	Ground truth - Comprehensive TTE - 2D and Doppler examinations for using commercial equipment (Philips Medical Systems) with S5-1 and X5-1 transducers<br><br>Non commercial AI algorithms - one based on

					multi 2d and doppler images, one based on single view with no doppler
94	Heart Failure	Firma et al.	756 participants with median age of 56 years	Sixteen nurses/nurse-assistants without prior experience in echocardiography underwent a 2-day hands-on intensive training to learn how to assess PLAX views<br><br>Cardiologists for confirmations	Philips Lumify Ultrasonography device for plax view detection<br><br>FoCUS assised AI - image interpretation by US2.AI
95	Heart Failure	Goto et al.	Multi-center study - 4,117 videos in the interal test group, hundreds of videos for each external validation test site.	Echocardiography lab sonographers	Comprehensive TTE<br><br>Non commercial AI algorithm using A4C view only
96	Heart Failure	Karra N et al.	12,281 patients with mean age of 72	Echocardiography lab sonographers	Comprehensive TTE<br><br>aisap.ai AI model using PLAX and A4C view only

97	Heart Failure	Oikonomou et al.	33,127 patients at Yale-New Haven Health System<br><br>5,624 patients at Mount Sinai Health System	Model development - certified sonographers and interpreted by certified cardiologists<br><br>Modeal testing - emergency room scans	Model development - comprehensive TTEs<br><br>Model testing- on data acquired by compact mid-range ultrasonography systems (ie, Sparq Ultrasound system, Philips Healthcare, Andover, MA, USA in the YNHHS)<br><br>Non commercial AI algorithm
102	Pulmonary Hypertension	Yu Jia Ke et al.	-	-	9 PoCUS studies
103	Pulmonary Hypertension	Binder et al.	-	9 novice physician sonographers (less than 50 echocardiographic exams)	PoCUS with color and continuous wave doppler
